# Effectiveness of modular approach in ensuring data quality in large-scale surveys: Evidence from National Family Health Survey – 4 (2015–2016)

**DOI:** 10.1016/j.ssmph.2022.101254

**Published:** 2022-10-04

**Authors:** Shri Kant Singh, Santosh Kumar Sharma, Md Juel Rana, Akash Porwal, Laxmi Kant Dwivedi

**Affiliations:** a Department of Survey Research & Data Analytics, International Institute for Population Sciences, Mumbai, India; b International Institute for Population Sciences, Mumbai, India; c G. B. Pant Social Science Institute, Allahabad, India; d Population Council, India

**Keywords:** Data quality, Modular approach, Non-sampling error, Skipping, Translator, NFHS

## Abstract

This study aims to examine the effect of administration of shorter and longer versions of questionnaires on key indicators such as age displacement, birth displacement, age heaping, and skipping questions on antenatal care (ANC) visits and use of contraceptive methods in India using National Family Health Survey (NFHS)-4 data. At the individual level, the effect of the adoption of the shorter and longer versions of the questionnaires on the age displacement of women and children and skipping of the key questions is insignificant. However, the results from the two-level logistic regression model reveal that at the primary sampling unit (PSU) level, work pressure, depending on the number of eligible women in a household, emerges as a confounder in skipping certain questions, namely ANC [1.18 (p < 0.09)] and contraceptive use [AOR = 1.17 (p < 0.05)]. To expand the coverage of NFHS in providing state- and district-level estimates since 2015, the overall sample size was increased from 88,562 households and 89,777 eligible women in 1992–93 to 6,01,509 households and 6,99,686 eligible women in 2015–16. As a strategy to reduce workload and non-sampling errors during the survey, a nested design and modular approach were adopted to provide estimates of maternal and child health indicators at the district/state level and sexual behaviour, HIV/AIDS, and women's empowerment at the state level. It was hypothesised that a longer version of the questionnaire canvassed in the state module may be detrimental to data quality issues. The findings of this study establish the effectiveness of adopting a modular approach in large-scale surveys, depending on the scale of investigation. However, the differential workload calls for expanding the duration of surveys in PSUs, where the number of eligible women is higher. State level variation in the key data quality indicators may be partially explained by differentials in the training of investigators by the agency and use of translators.

## Highlights

• Adoption of modular approach in NFHS does not have any relationship with data quality.
• PSU level pressure on investigators is associated with the skipping of key questions.
• There is significant variation in key skipping questions across the states of India.
• Birth and age displacement are also considerably varied across the states of India.

## 1 Introduction

Large-scale sample surveys are an important source of data on various demographic and health-related indicators in a country. In developing countries, particularly those collecting retrospective data, these surveys have traditionally suffered from incomplete and inconsistent reporting (Croft, 1991). The quality of the data was significantly affected by both sampling and non-sampling errors. Among non-sampling errors, respondents’ under-reporting of events, incorrect recording of information by the interviewer, errors arising from questionnaire design, etc., are of a more serious nature. Errors due to non-response arise when some units do not respond or are not investigated at all. In addition, an increase in the length of the questionnaire can increase interview time and ultimately be more burdensome for both the interviewer and the respondent.

Age is one of the most important demographic variables that is rarely free from reporting bias in developing countries, including India. Age misreporting is a common phenomenon in demographic and health surveys for which there are numerous reasons. The trend and pattern of disparity in age heaping varies between countries (Blum & Krauss, 2018; Fayehun et al., 2020; Singh, 2017; Singh et al., 2021). Data collected through census or sample surveys in developing countries are more likely to have age misreporting than data collected in developed countries (Talib et al., 2010; Singh et al., 2021).

One of the most commonly mentioned problems of demographic and health surveys (DHS) birth histories is the displacement of births, meaning that birth dates are moved from one calendar year to another (Arnold, 1990; Pullum, 2006, pp. 1985–2003) (Schoumaker, 2011). This birth displacement may also be the result of both reporting issues due to recall bias and intentional incorrect entry by field investigators to avoid a lengthy questionnaire (Al Zalak, 2017).

Several studies documented that age and date of birth remain the main focus of data quality issues (Lyons-Amos & Stones, 2017; Pullum, 2006, pp. 1985–2003; Pullum, 2019; Pullum, 2017) An analysis of age heaping over time in 34 countries in Sub Saharan Africa between 1987 and 2015 concluded that a number of countries have considerable increases in the proportion of age misreported (Lyons-Amos & Stones, 2017). Some studies focused on fieldwork conditions and interviewer characteristics to better understand data quality (Johnson et al., 2009; Pullum, 2018) suggesting that older, more educated, and experienced interviewers are more likely to collect high-quality data.

The scope and complexity of DHS have increased since it extended the length of the questionnaire and additional survey modules. Bradley (2016) and Short Fabic et al. (2012) concluded that a longer questionnaire duration is significantly associated with a decrease in data quality. Allen et al. (2020) argued that longer questionnaires would have different effects than shorter versions of questionnaires on fieldwork, interviewer fatigue, performance, and survey implementation. It offers an opportunity to explore the extent to which questionnaire length may have an effect on data quality. An increase in questionnaire length can increase interview time and may ultimately present a greater burden for both the interviewer and the respondent (Sbort Fabic et al., 2012; Allen et al., 2020). Many DHS surveys include modules or additional topic-specific sections only among subsamples, resulting in questionnaires of different lengths within the same survey. Individual interviews vary in length because respondents are asked fewer or more questions based on their circumstances (Allen et al., 2020). Several studies have shown that large-scale population-based surveys are facing a persistent decrease in the response rate due to the effect of the length of the questionnaire (Wallander et al., 2015; Morton, Cahill & Hartge, 2006; Deutskens et al., 2004; Bouranta, Chitris, & Paravantis, 2009; Blumenberg et al., 2019).

This study has been conceptualised with the following rationale. First, NFHS surveys are representative of the population at the national, state, and district levels (since 2015), and the overall high quality of the survey data has given policymakers and programme planners confidence in using NFHS data as a key source (often the only source) of relevant data. Since NFHS-4, district-level estimates were also provided, as the sample size increased by six times from the first three rounds of NFHS. Second, with each successive round, additional components were added to capture the newly initiated health and development programmes by the government. These additions have resulted in a substantial increase in survey duration depending on age, marital status, and the number of children in the five years preceding the survey. Third, among the different sources of sampling and non-sampling errors, the most commonly encountered error was inaccurate age reporting. Many demographic and health estimates are age specific, such as estimates of age-specific fertility rates and infant and child mortality rates, which can be affected by the misreporting of ages and dates of birth and/or death for a woman and her children. The age displacement of children can seriously distort estimates of current levels and recent trends in fertility and mortality and is by no means unique to DHS surveys. Evaluation of censuses and community surveys has revealed severe age misreporting (Bailey, 1996; Mukherjee, 1988). In this context, this study aims to examine the effectiveness of administering a shorter and longer version of a questionnaire on key data quality indicators.

The study may provide evidence of improvement in data quality despite continuously increasing contents and coverage of NFHS in India, which have enhanced challenges in survey implementation and may also have a detrimental effect on data quality. One of the tools for reducing the burden of the large content of the questionnaire is the introduction of the state module in the sample design. Since the NFHS-4 has adopted this design for the first time, the effects of the introduction of a nested design and modular approach in sampling design on the key demographic and health care indicators (skipping questions) need to be studied.

## 2 Materials and methods

### 2.1 Data

This study used data from NFHS-4 (2015–16). In this survey, the challenges of expanding the content and coverage of NFHS resulted in innovations in data collection by adopting various strategies of survey implementation. These innovations are mentioned in Supplementary Table S1. A major one was the introduction of a modular approach to the survey. In each district of India, approximately 30% of the primary sampling units (PSUs) were randomly selected for implementation of the longer version of the questionnaire. In the selected PSUs, every alternate household was chosen for the administration of the longer version. This research on data quality is based only on those PSUs that were selected for canvassing longer versions of questionnaires in 50% of the selected households and shorter versions in the remaining selected households.

The analyses were performed using the sample only for the PSUs that were selected for the state module. The sample selection criteria for each outcome indicator are presented in Table 1. Thus, in the selected sample, ideally, the sample size in the state and district modules should be equally divided because the state module is administered in every second household in each selected PSU.

**Table 1:** Sample selection criteria for each outcome variables included in this study.

Outcome indicators	Measures	Sample selection criteria	Sample distribution
Age displacement	14/15 ratio	The women aged 14 and 15 years	Table S5
	50/49 ratio	The women aged 50 and 49 years	Table S5
Birth displacement	5/6 ratio	The children aged 5 and 6 years	Table S6
Age heaping or digit preference	Whipple's index	The Women aged 18–47 years	Table S3
	Myers' blended index	The Women aged 20–49 years	Table S4
Skipping of questions	No ANC	The women aged 15–49 years who have delivered at least one birth in last five years of the survey	Table S7
	No contraceptive use	The currently married women aged 15–49 years	Table S7

### 2.2 Variables

#### 2.2.1 Outcome variables

The key outcome variables were age displacement, birth displacement, age heaping, skipping questions on antenatal care (ANC) visits, and the use of contraceptive methods. For comparison purposes between the state and district modules, state-level analyses were carried out on the outcome indicators and are presented in the graphs. A detailed description of the outcome indicators is provided below:

### 2.3 Methodology

The methods used in the analyses included the ratio of initial and terminal age, ratio of survey years, Myer's index, Whipple index, two-stage logistic regression, and propensity matching score. A brief description of each is provided below.

a) **Age displacement**: The eligible ages for men and women were 15–54 and 15–49 years, respectively. To identify age displacement, the age ratio (14 years/15 years) was estimated for women and men in states and union territories in India.
b) **Birth displacement:** To identify the birth displacement in the data, the ratio between children born in the six and five years preceding the survey.
c) **Age heaping or digit preference:** Whipple's index and Myers' blended index were used to evaluate the extent of inaccuracy in terms of age heaping and digit preference. Age heaping or age *preference* is the tendency of individuals to incorrectly report their age or date of birth. Individuals' heaping behaviours favour certain ages, commonly those ending in ‘0’ or ‘5’.
d) **Skipping questions**: There are different instructions in terms of filter skipping, which gives the researcher an opportunity to capture authentic information. To understand the skip pattern of selected indicators with a larger skip from NFHS-3 to NFHS-4, the percentage of women who said no to the selected indicators in both surveys will be estimated. The specific indicators did not undergo any antenatal check-ups and did not use any contraceptive methods. Here, it is important to note that the skipping of questions includes both intentional and unintentional skipping from both the respondents' and investigators' perspectives.

A two-level logistic regression model was applied for three indicators: digit preference (age heaping) at 0 and 5 years, no ANC visits, and not using contraceptive methods. In these two-level logistic regression analyses, the pressure of the state module was incorporated by combining the proportion of households with eligible women in a PSU, average number of eligible women per household in the PSU, and proportion of women to whom the state module was administered. Details of the multilevel analysis are provided in the supplementary file.

Furthermore, propensity score matching (PSM) analysis was carried out considering the state module as the intervention group for the three outcome variables. In the selected PSUs, to administer the state module, a questionnaire with state module was administered to each alternative household. Given the similar socioeconomic characteristics among households within a PSU, the administration of a questionnaire with a state module (longer version of the questionnaire) would work as an intervention. Thus, the effects of the modular approach on reporting quality, particularly age heaping and skipping questions, can be measured using PSM analysis.

## 3 Results

### 3.1 Age displacement

**Fig. 1:**
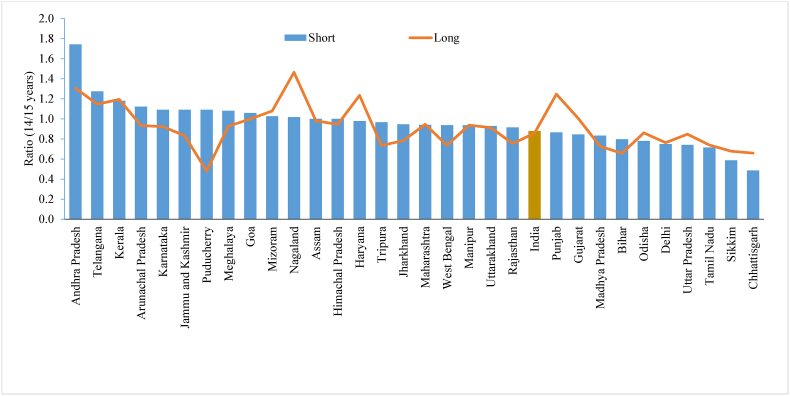
Ratio between reported number of women at age 14 and 15 by shorter and longer version of the questionnaire across the states of India, 2015-16 Note: States and union territories with less than 30 sample size is dropped.

**Fig. 2:**
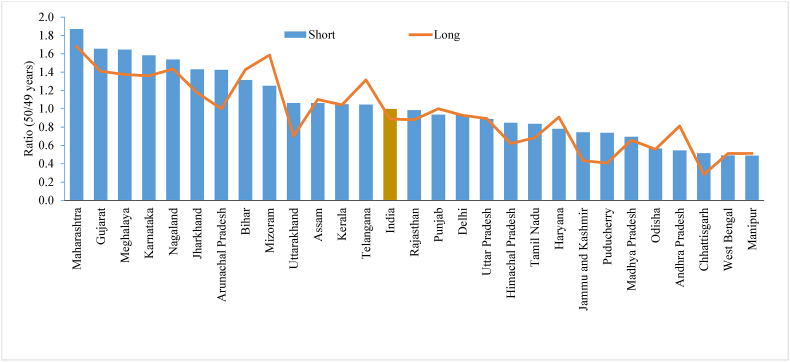
Ratio between reported number of women at age 50 and 49 by shorter and longer version of the questionnaire across the states of India, 2015-16 Note: States and union territories with less than 30 sample size is dropped.

Fig. 1 shows the differences in age displacement among women in the shorter and longer versions of the questionnaire by taking the ratio of the reported number of women aged 15 and 14 across states/UTs in India. A value close to 1 indicates a smaller degree of age displacement, while values less than 1 indicate downward displacement, and values greater than 1 indicate upward displacement. With the existing age-sex composition of the population, the age ratio may vary from 0.90 to 1.1; therefore, values of the ratio in this range are well accepted. There was no evidence of age displacement at the lower end among women in the shorter and longer versions of the questionnaire in India. The age displacement was higher in Andhra Pradesh and Telangana in the shorter version of the questionnaire than in the longer version, whereas Mizoram, Nagaland, Haryana, Punjab, and Gujarat showed higher age displacement in the longer version of the questionnaire. Similarly, age displacement was slightly higher at the upper end (50/49) in the shorter version than in the longer version of the questionnaire in India. The displacement of the age of women was higher in Maharashtra, Gujarat, Meghalaya, Karnataka, Nagaland, Jharkhand, Bihar, and Mizoram (Fig. 2).

### 3.2 Birth displacement

**Fig. 3:**
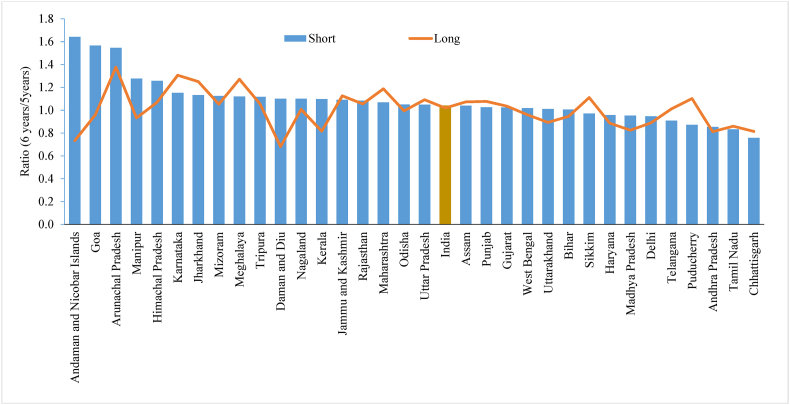
Ratio between reported number of children aged 6 years and 5 years by shorter and longer version of the questionnaire across the states of India, 2015-16 Note: States and union territories with less than 30 sample size is dropped.

Several maternal and child health indicators are derived using information on the date of birth based on a five-year reference period, wherein there may be a tendency to shift the date of birth by a few investigators to reduce their workload. Fig. 3 shows the ratio of the reported number of children aged six and five years using the shorter and longer versions of the questionnaire across the states of India. The figure shows that displacement is higher in some states, such as the Andaman and Nicobar Islands (1.64), Goa (1.57), Arunachal Pradesh (1.55), Manipur (1.28), and Himachal Pradesh (1.26) in the shorter version of the questionnaire than the longer version. Additionally, the majority of the states reported children under the age of 5 years than in the shorter version of the questionnaire.

### 3.3 Age heaping

**Fig. 4:**
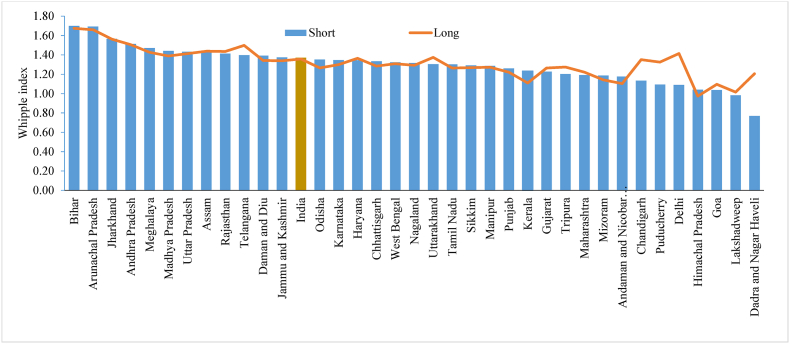
Whipple index by shorter and longer version of the questionnaire across the states of India, 2015-16.

During fieldwork, when either the respondent or interviewer estimated an unknown age or year, the tendency was to select numbers ending in 0 or 5, resulting in heaping on these digits. We used two measures to evaluate the extent of inaccuracy (in terms of age heaping and digit preference) in age reporting: Whipple's and Myers' blended indices. Fig. 4 shows the Whipple index for selected Indian states/UTs using the shorter and longer versions of the questionnaire. This shows that the value of the Whipple index is similar in the shorter and longer versions of the questionnaire across most states in India. However, the value of the Whipple index revealed that age was not correctly reported in the majority of the states, as the value was higher than the threshold limit (110–125) suggesting that digit preferences of 0 and 5 are higher in many states. These states were Bihar, Andhra Pradesh, Madhya Pradesh, Uttar Pradesh, Assam, Rajasthan, Odisha, and Haryana.

**Fig. 5:**
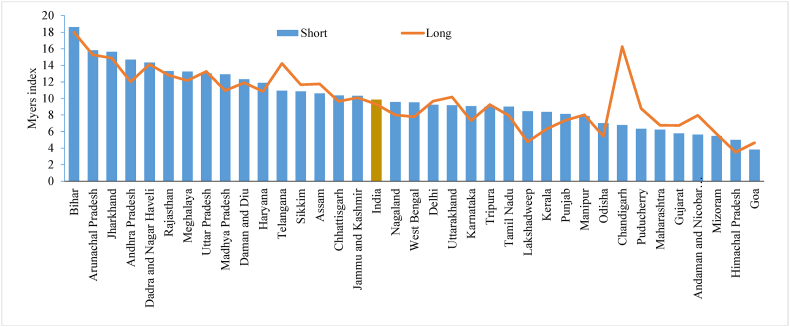
Myers blended index by shorter and longer version of the questionnaire across the states of India, 2015-16.

Similarly, Fig. 5 reports the age heaping measured by the Myers blended index using the shorter and longer versions of the questionnaire across the states of India. The figure shows that the value of Myers' blended index is almost similar in the shorter and longer versions of the questionnaire across the states, except for a few states such as Telangana and Chandigarh. Overall, the value of Myer's blended index is 9.8 in the shorter and 9.3 in the longer version of the questionnaire. The value of Myer's index varies from 3.8 to 18.6 in the shorter version of the questionnaire, while in the longer version, it ranges from 3.5 to 18.0 across the states/UTs.

Table 2 presents the results of a two-level multilevel logistic regression of reporting age ending with 0 and 5 among women aged 18–47 years in India, 2015–16. The level of variation in age heaping between communities was similar in Model 1 (null model) and Model 2 (individual/household level characteristics), as the value of intra-class correlation coefficient (ICC) 2% of the total variance in age heaping was attributable to differences across communities. After including community-level factors in Model 3, the ICC values decreased to 1.4%. Further, there was no significant difference in age misreporting/heaping in the long and short versions of the questionnaire in Models 2 and 3. The likelihood of age heaping is higher among households with multiple eligible women. As the age of women increased, the likelihood of age ending with 0 and 5 increased with reference to the population aged 15–24 years in models 2 and 3. In addition, as the wealth index increased, the likelihood of age heaping decreased significantly in both models. Women belonging to the Muslim religion, SC, ST, and OBCs castes are more likely to report age ending with 0 and 5 than their counterparts. The pressure of the state module was not significantly associated with age heaping among women. Most states showed a insignificant association with age heaping among women.

**Table 2:** Results from two level logistic regression model: Estimated adjusted odds ratios of reporting age ending with 0 and 5 among the women aged 18–47 years in India, 2015-16.

Background variables	Model 1	Model 2	Model 3
***Individual/household level factors***			
**Modules**			
District module®	─	1.00	1.00
State module	─	1.01 (0.99–1.04)	1.01 (0.98–1.03)
**Multiple visits to the households**			
Single ®	─	1.00	1.00
Multiple	─	1 (0.96–1.03)	1 (0.97–1.03)
**Use of translator**			
No®	─	1.00	1.00
Yes	─	0.99 (0.93–1.07)	1.01 (0.94–1.08)
**Number of eligible women in the household**			
Single®	─	1.00	1.00
Multiple	─	1.06 (1.04–1.08)***	1.04 (1–1.07)*
**Age group of women**			
15-25®	─	1.00	1.00
25–29	─	1.91 (1.84–1.97)***	1.93 (1.86–1.99)***
30–34	─	2.51 (2.42–2.59)***	2.55 (2.47–2.64)***
35–39	─	2.5 (2.42–2.59)***	2.57 (2.48–2.66)***
40–49	─	3.73 (3.61–3.85)***	3.86 (3.74–3.99)***
**Wealth quintile**			
Poorest®		1.00	1.00
Poorer	─	0.91 (0.88–0.94)***	0.94 (0.91–0.97)***
Middle	─	0.85 (0.82–0.89)***	0.89 (0.86–0.93)***
Richer	─	0.76 (0.73–0.79)***	0.8 (0.77–0.84)***
Richest	─	0.68 (0.65–0.71)***	0.72 (0.69–0.76)***
**Level of education**			
No education ®	─	1.00	1.00
Primary	─	0.98 (0.95–1.02)	1.01 (0.98–1.05)
Secondary	─	1.01 (0.98–1.04)	1.05 (1.02–1.08)**
Higher	─	1.15 (1.1–1.2)***	1.19 (1.14–1.25)***
**Access to mass media**			
No®	─	1.00	1.00
Yes	─	0.94 (0.91–0.97)***	0.97 (0.94–1)
**Current work status**			
Not working ®	─	1.00	1.00
Working	─	0.89 (0.86–0.92)***	0.9 (0.87–0.93)***
**Religion**			
Hindu ®	─	1.00	1.00
Muslims	─	1.15 (1.11–1.19)***	1.16 (1.12–1.2)***
Others	─	0.99 (0.95–1.03)	0.97 (0.93–1.02)
**Caste**			
General®	─	1.00	1.00
SC	─	1.1 (1.06–1.13)***	1.09 (1.05–1.13)***
ST	─	1.05 (1.01–1.09)*	1.06 (1.02–1.1)**
OBC	─	1.09 (1.06–1.12)***	1.05 (1.02–1.08)***
***Community level factors***			
**Pressure of eligible women and state module**^a^			
Low ®	─	─	1.00
Medium	─	─	0.98 (0.95–1.01)
High	─	─	1.02 (0.98–1.07)
**Place of residence**			
Urban ®	─	─	1.00
Rural	─	─	1.02 (1–1.05)
**States**			
Jammu & Kashmir ®	─	─	1.00
Andhra Pradesh	─	─	1.21 (1.09, 1.34)***
Arunachal Pradesh	─	─	1.39 (1.26, 1.53)***
Assam	─	─	1.04 (0.96, 1.11)
Bihar	─	─	1.35 (1.26, 1.44)***
Chhattisgarh	─	─	0.98 (0.9, 1.06)
Goa	─	─	0.77 (0.66, 0.89)***
Gujarat	─	─	0.93 (0.86, 1)*
Haryana	─	─	1.16 (1.06, 1.26)***
Himachal Pradesh	─	─	0.69 (0.63, 0.75)***
Jharkhand	─	─	1.24 (1.15, 1.33)***
Karnataka	─	─	0.97 (0.9, 1.04)
Kerala	─	─	0.82 (0.75, 0.9)***
Madhya Pradesh	─	─	1.1 (1.03, 1.17)***
Maharashtra	─	─	0.89 (0.82, 0.96)***
Manipur	─	─	0.92 (0.83, 1.02)
Meghalaya	─	─	1.19 (1.05, 1.33)*
Mizoram	─	─	0.88 (0.78, 0.98)*
Nagaland	─	─	0.96 (0.85, 1.07)
Delhi	─	─	1.06 (0.92, 1.21)
Odisha	─	─	0.93 (0.86, 1)
Punjab	─	─	1.01 (0.93, 1.11)
Rajasthan	─	─	1.16 (1.08, 1.25)***
Sikkim	─	─	1 (0.87, 1.15)
Tamil Nadu	─	─	0.93 (0.86, 1)*
Tripura	─	─	0.84 (0.74, 0.96)*
Uttar Pradesh	─	─	1.11 (1.05, 1.18)***
Uttarakhand	─	─	1.06 (0.97, 1.16)
West Bengal	─	─	0.95 (0.87, 1.03)
Telangana	─	─	1.17 (1.04, 1.31)*
Constant	0.37 (0.37–0.37)***	0.2 (0.19–0.21)***	0.14 (0.12–0.17)***
Random effects variance	0.07 (0.06–0.08)	0.07 (0.06–0.08)	0.05 (0.04–0.06)
ICC (in 100)	2.0 (1.8–2.3)	2.0 (1.7–2.2)	1.4 (1.2–1.7)
Number of observations	2,09,904	2,09,904	2,09,904
Wald test X2	─	8136.0	8695.5
LR test vs. logistic regression: Chi^2^	332.19	288.77	162.57
Log likelihood	−122890.87	−118496.6	−118188.05

Note: Ref. stands for reference group of the independent variables.

***p < 0.001, **p < 0.01, *p < 0.05.

a Pressure of state module is the tertile of score generated by multiplying the proportion of the household which have eligible women in a PSU, average number of eligible women per household in the PSU and the proportion of women to whom state module is administered.

### 3.4 Skipping questions

#### 3.4.1 No antenatal care visits

**Fig. 6:**
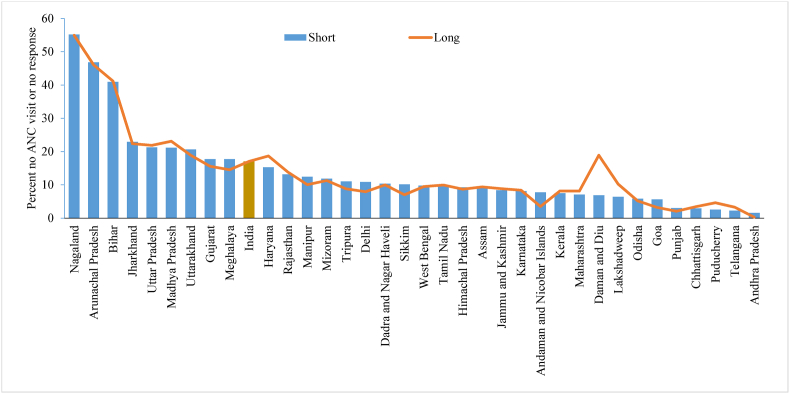
Percentage of women reported no ANC visit (ANC) during the pregnancy of their last birth who born in last five years by shorter and longer version of the questionnaire across the states of India, 2015-16.

In every survey, there were different instructions in terms of filters and skips that provided the researcher with the opportunity to capture authentic information. Fig. 6 displays the percentage of women who did not report ANC visits during their last pregnancy in the five years preceding the survey. The figure shows that overall, at the national level, majority of states have similar reporting of “No” response to any ANC visits in the shorter and longer version of the questionnaire.

Table 3 presents the results of two-level multilevel logistic regression of ‘No response’ to ANC visits among women who delivered in the last five years preceding the survey in 2015–16. The results showed no significant association between the state module and non-response to ANC visits. The likelihood of ‘No response’ to ANC visits was higher among those households with multiple eligible women in model 2 [AOR = 1.07; 95% CI: 1.02–1.13]. As the age of women increases, the likelihood of non-response to ANC visits increases with reference to age 15–24 in both Models 2 and 3. As the wealth index and schooling of women increased, the likelihood of non-response to ANC visits decreased significantly in both the models. Women belonging to other religions and castes, such as the ST caste, are more likely to report non-response to ANC visits than their counterparts. The high pressure of the state module is significantly 1.18 (p < 0.09) times more likely to report non-response to the ANC visit question.

**Table 3:** Results from two level logistic regression model: Estimated adjusted odds ratios of no antenatal care visits or nonresponse among the women who delivered their last birth in last five years in India, 2015-16.

Background variables	Model 1	Model 2	Model 3
***Individual/household level factors***			
**Modules**			
District module®	─	1.00	1.00
State module	─	0.95 (0.89–1.01)	0.95 (0.89–1.01)
**Multiple visits to the household**			
Single®	─	1.00	1.00
Multiple	─	1 (0.93–1.09)	1 (0.92–1.08)
**Use of translator**			
No®	─	1.00	1.00
Yes	─	1.14 (0.95–1.37)	1.29 (1.08–1.55)**
**Number of eligible women in the household**			
Single®	─	1.00	1.00
Multiple	─	1.07 (1–1.13)*	0.93 (0.84–1.04)
**Age group of women**			
15-25®	─	1.00	1.00
25–29	─	1.15 (1.08–1.23)***	1.15 (1.08–1.23)***
30–34	─	1.39 (1.28–1.5)***	1.38 (1.27–1.49)***
35–39	─	1.58 (1.43–1.75)***	1.58 (1.43–1.74)***
40–49	─	2.45 (2.14–2.81)***	2.46 (2.14–2.81)***
**Wealth quintile**			
Poorest ®		1.00	1.00
Poorer	─	0.73 (0.67–0.78)***	0.74 (0.68–0.8)***
Middle	─	0.51 (0.47–0.56)***	0.53 (0.49–0.59)***
Richer	─	0.37 (0.33–0.41)***	0.4 (0.35–0.45)***
Richest	─	0.25 (0.22–0.29)***	0.28 (0.25–0.33)***
**Level of education**			
No education ®	─	1.00	1.00
Primary	─	0.67 (0.62–0.73)***	0.69 (0.64–0.75)***
Secondary	─	0.52 (0.48–0.56)***	0.55 (0.51–0.59)***
Higher	─	0.36 (0.31–0.41)***	0.37 (0.32–0.42)***
**Access to mass media**			
No®	─	1.00	1.00
Yes	─	0.63 (0.59–0.68)***	0.7 (0.65–0.75)***
**Current work status**			
Not working ®	─	1.00	1.00
Working	─	1 (0.91–1.1)	1.02 (0.92–1.12)
**Religion**			
Hindu®	─	1.00	1.00
Muslims	─	0.97 (0.88–1.07)	1.05 (0.94–1.16)
Others	─	1.47 (1.29–1.67)***	1.08 (0.92–1.27)
**Caste**			
General ®	─	1.00	1.00
SC	─	1.14 (1.03–1.27)**	1.05 (0.95–1.16)
ST	─	1.26 (1.12–1.41)***	1.21 (1.07–1.36)**
OBC	─	1.18 (1.08–1.28)***	1.02 (0.94–1.12)
***Community level factors***			
**Pressure of eligible women and state module**^a^			
Low®	─	─	1.00
Medium	─	─	1.04 (0.95–1.14)
High	─	─	1.18 (1.02–1.36)*
**Place of residence**			
Urban ®	─	─	1.00
Rural	─	─	1.09 (0.97–1.21)
**States**			
Jammu & Kashmir®	─	─	1.00
Andhra Pradesh	─	─	0.11 (0.05, 0.28)***
Arunachal Pradesh	─	─	16.84 (12.06, 23.5)***
Assam	─	─	0.93 (0.69, 1.26)
Bihar	─	─	9.35 (7.24, 12.07)***
Chhattisgarh	─	─	0.28 (0.18, 0.41)***
Goa	─	─	1.04 (0.51, 2.12)
Gujarat	─	─	3.56 (2.71, 4.7)***
Haryana	─	─	3.79 (2.71, 5.3)***
Himachal Pradesh	─	─	2.36 (1.65, 3.39)***
Jharkhand	─	─	3.03 (2.29, 4.01)***
Karnataka	─	─	1.5 (1.08, 2.08)*
Kerala	─	─	2.65 (1.77, 3.97)***
Madhya Pradesh	─	─	3.93 (3.05, 5.06)***
Maharashtra	─	─	1.27 (0.92, 1.75)
Manipur	─	─	1.33 (0.9, 1.97)
Meghalaya	─	─	1.93 (1.25, 2.96)***
Mizoram	─	─	1.71 (1.12, 2.61)*
Nagaland	─	─	23.09 (15.94, 33.45)***
Delhi	─	─	3.15 (1.8, 5.53)***
Odisha	─	─	0.53 (0.38, 0.73)***
Punjab	─	─	0.55 (0.34, 0.91)*
Rajasthan	─	─	1.84 (1.4, 2.43)***
Sikkim	─	─	1.5 (0.8, 2.8)
Tamil Nadu	─	─	2.57 (1.9, 3.46)***
Tripura	─	─	1.47 (0.87, 2.5)
Uttar Pradesh	─	─	3.56 (2.81, 4.52)***
Uttarakhand	─	─	5.74 (4.13, 7.96)***
West Bengal	─	─	1.32 (0.92, 1.89)
Telangana	─	─	0.44 (0.23, 0.84)*
Constant	0.08 (0.07–0.08)***	0.23 (0.2–0.26)***	0.06 (0.02–0.16)***
Random effects variance	3.39 (3.18–3.62)***	2.54 (2.37–2.72)***	1.8 (1.67–1.94)***
ICC (in 100)	50.8 (49.1–52.4)***	43.6 (41.9–45.3)***	35.4 (33.7–37.1)***
Number of observations	66,028	66,028	66,028
Wald test X2	─	2957	4275
LR test vs. logistic regression: Chi2	8674	5429	3540
Log likelihood	−25,806	−24,211	−23,315

Note: Ref. stands for reference group of the independent variables.

***p < 0.001, **p < 0.01, *p < 0.05.

a Pressure of state module is the tertile of score generated by multiplying the proportion of the household which have eligible women in a PSU, average number of eligible women per household in the PSU and the proportion of women to whom state module is administered.

Most states showed a insignificant association with age heaping among women. However, there are few states that show the significant and higher likelihood of ‘No response’ to ANC visits reported by women, which are Arunachal Pradesh, Bihar, Gujrat, Haryana, Madhya Pradesh, Uttar Pradesh, Rajasthan, and Tamil Nadu. Furthermore, the level of variation in ‘no response’ to ANC visits between communities is decreasing from model 1 (null model) to model 2 (individual/household level characteristics) to model 3 (community level). 51% of the total variance in the ‘no response’ to ANC visits can be attributed to the differences across communities in Model 1. When individual- and household-level factors were integrated into the null model, the ICC value decreased to 44% in model 2. With the integration of community-level factors in Models 1 and 2, ICC values decreased to 35%.

#### 3.4.2 Currently not using any contraceptive methods

**Fig. 7:**
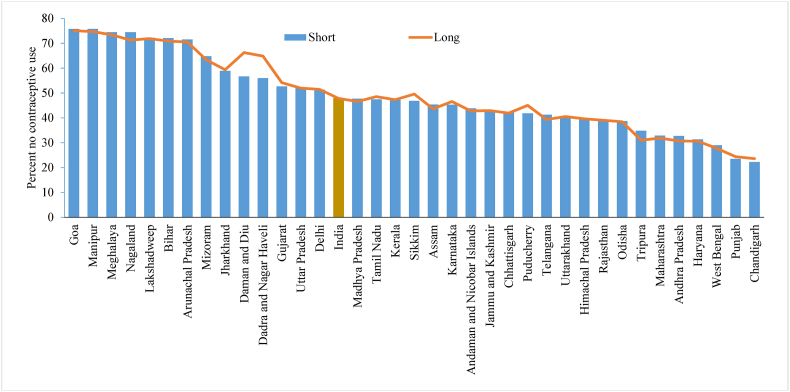
Percentage of women who did not using any contraceptive methods by shorter and longer version of the questionnaire across the states of India, 2015-16.

Fig. 7 presents the percentage of women who did not report the current use of any contraception method in both the short and long versions of the questionnaire during the NFHS-4. It is evident that there is no significant difference in the reporting of ‘no response’ to using any contraceptive method by both versions of the questionnaire, except for some smaller states. The percentage of women who reported no response to currently using a family planning method in either type of questionnaire was nearly the same (longer version, 47.7%; shorter version, 47.8%].

Table 4 presents the results of the two levels of not using contraceptive methods among currently married women in India, 2015–16. The longer version of the questionnaire (state module) was more likely to report not using any contraceptive methods among currently married women in both Model 2 [AOR = 1.03; 95% CI: 1.01–1.06] and Model 3 [AOR = 1.04; 95% CI: 1.01–1.06]. Multiple visits to households were significantly associated with and less likely to report not using contraceptive methods among women, indicating that multiple visits to households decreased the skipping of questions regarding the use of contraceptive methods. It was found that the use of a translator during interviews increases the likelihood of skipping the question of the use of contraception methods among women in both models. The likelihood of not using contraceptive methods was higher among households that had multiple eligible women in both model 2 [AOR = 1.17; 95% CI: 1.14–1.20] and model 3 [AOR = 1.07; 95% CI: 1.02–1.12]. In the case of the wealth quintile, as the wealth index increases, the likelihood of skipping the question of contraceptive methods decreases significantly in both models. With an increase in the level of education from primary to higher, the likelihood of skipping contraceptive questions increases among women in both models. Women belonging to Muslim and other religions and castes, such as the ST caste, are more likely to report non-response to contraception questions than their counterparts. Medium and high pressure of the state module on female respondents showed a higher likelihood of skipping the contraceptive questions.

**Table 4:** Results from two level logistic regression model: Estimated adjusted odds ratios of not using contraception among the currently married women in India, 2015-16.

Background variables	Model 1	Model 2	Model 3
***Individual/household level factors***			
**Modules**			
District module®	─	1.00	1.00
State module	─	1.03 (1.01–1.06)*	1.04 (1.01–1.06)*
**Multiple visits to the households**			
Single®	─	1.00	1.00
Multiple	─	0.95 (0.92–0.99)*	0.96 (0.92–0.99)*
**Use of translator**			
No®	─	1.00	1.00
Yes	─	1.26 (1.16–1.38)***	1.28 (1.18–1.4)***
**Number of eligible women in the household**			
Single®	─	1.00	1.00
Multiple	─	1.17 (1.14–1.2)***	1.07 (1.02–1.12)**
**Age group of women**			
15-25®	─	1.00	1.00
25–29	─	0.33 (0.32–0.35)***	0.33 (0.32–0.35)***
30–34	─	0.17 (0.17–0.18)***	0.17 (0.17–0.18)***
35–39	─	0.13 (0.12–0.14)***	0.13 (0.12–0.13)***
40–49	─	0.17 (0.16–0.18)***	0.17 (0.16–0.17)***
**Wealth quintile**			
Poorest®		1.00	1.00
Poorer	─	0.86 (0.82–0.89)***	0.86 (0.83–0.9)***
Middle	─	0.79 (0.76–0.83)***	0.8 (0.76–0.84)***
Richer	─	0.77 (0.73–0.81)***	0.79 (0.75–0.83)***
Richest	─	0.74 (0.7–0.78)***	0.8 (0.75–0.85)***
**Level of education**			
No education®	─	1.00	1.00
Primary	─	0.92 (0.89–0.96)***	0.91 (0.88–0.95)***
Secondary	─	1.11 (1.07–1.14)***	1.08 (1.04–1.11)***
Higher	─	1.72 (1.63–1.81)***	1.65 (1.57–1.74)***
**Access to mass media**			
No®	─	1.00	1.00
Yes	─	0.74 (0.71–0.77)***	0.76 (0.73–0.78)***
**Current work status**			
Not working®	─	1.00	1.00
Working	─	0.78 (0.75–0.81)***	0.77 (0.74–0.8)***
**Religion**			
Hindu®	─	1.00	1.00
Muslims	─	1.5 (1.43–1.57)***	1.53 (1.46–1.6)***
Others	─	1.44 (1.36–1.52)***	1.13 (1.06–1.2)***
**Caste**			
General®	─	1.00	1.00
SC	─	1.04 (1–1.09)	1.03 (0.99–1.08)
ST	─	1.33 (1.26–1.4)***	1.14 (1.08–1.19)***
OBC	─	1.04 (1–1.07)*	0.98 (0.94–1.01)
***Community level factors***			
**Pressure of eligible women and state module**^a^			
Low®	─	─	1.00
Medium	─	─	1.07 (1.02–1.11)**
High	─	─	1.15 (1.08–1.22)***
**Place of residence**			
Urban®	─	─	1.00
Rural	─	─	1.03 (0.98–1.08)
**States**			
Jammu & Kashmir®			1.00
Andhra Pradesh	─	─	0.66 (0.55, 0.8)***
Arunachal Pradesh	─	─	4.63 (3.91, 5.49)***
Assam	─	─	1.01 (0.89, 1.15)
Bihar	─	─	3.87 (3.42, 4.38)***
Chhattisgarh	─	─	1.04 (0.9, 1.2)
Goa	─	─	8.2 (6.34, 10.59)***
Gujarat	─	─	1.96 (1.73, 2.22)***
Haryana	─	─	0.6 (0.51, 0.69)***
Himachal Pradesh	─	─	1.17 (1.00, 1.36)
Jharkhand	─	─	1.97 (1.73, 2.26)***
Karnataka	─	─	1.38 (1.2, 1.58)***
Kerala	─	─	1.55 (1.33, 1.82)***
Madhya Pradesh	─	─	1.27 (1.14, 1.43)***
Maharashtra	─	─	0.64 (0.56, 0.73)***
Manipur	─	─	6.62 (5.52, 7.95)***
Meghalaya	─	─	5.24 (4.21, 6.52)***
Mizoram	─	─	3.37 (2.78, 4.09)***
Nagaland	─	─	4.96 (4.07, 6.04)***
Delhi	─	─	1.92 (1.52, 2.41)***
Odisha	─	─	0.9 (0.79, 1.03)
Punjab	─	─	0.46 (0.39, 0.54)***
Rajasthan	─	─	0.84 (0.74, 0.95)*
Sikkim	─	─	1.71 (1.35, 2.18)***
Tamil Nadu	─	─	1.79 (1.58, 2.03)***
Tripura	─	─	0.65 (0.52, 0.81)***
Uttar Pradesh	─	─	1.52 (1.36, 1.69)***
Uttarakhand	─	─	1.01 (0.86, 1.18)
West Bengal	─	─	0.42 (0.36, 0.49)***
Telangana	─	─	1.02 (0.84, 1.24)
Constant	0.92 (0.9–0.94)***	4.11 (3.86–4.37)***	3.94 (2.86–5.42)***
Random effects variance	0.93 (0.89–0.97)***	0.98 (0.94–1.03)***	0.63 (0.61–0.66)***
ICC (in 100)	22.1 (21.4–22.8)***	23.0 (22.3–23.8)***	16.2 (15.6–16.8)***
Number of observations	1,74,207	1,74,207	1,74,207
Wald test X2	─	15557.98	18189.88
LR test vs. logistic regression: Chi2	16266.88	14526.57	8328.63
Log likelihood	−112461.49	−103377.98	−101757.02

Note: Ref. stands for reference group of the independent variables.

***p < 0.001, **p < 0.01, *p < 0.05.

a Pressure of state module is the tertile of score generated by multiplying the proportion of the household which have eligible women in a PSU, average number of eligible women per household in the PSU and the proportion of women to whom state module is administered.

State-wise analysis portrays that the skipping of contraceptive question in Andhra Pradesh, Haryana, Maharashtra Odisha, Punjab, Rajasthan, Tripura, and West Bengal is less likely (p < 0.001) but is higher as compared to the Jammu & Kashmir. Furthermore, the multilevel model applied without covariates (null model), on not using contraceptive methods among currently married women, showed a significant amount of variation in the use of contraceptive methods across individuals/households and communities. Based on the ICC value, 22% of the total variance in skipping the contraceptive question was attributable to differences across communities in model 1 (null model). When individual- and household-level factors were integrated into the null model, the ICC value increased slightly to 23% in Model 2. Similarly, when community-level factors were integrated with the factors included in Models 1 and 2, the ICC values decreased to 16%.

Supplementary Table S8 presents the results of propensity score matching (PSM) for age heaping and skipping questions on ANC and contraceptive use. In this analysis, the longer version of the questionnaire was considered the intervention group, while the shorter version of the questionnaire was considered the control group. It is evident from the results that the values of average treatment on the treated (ATT), average treatment on the untreated (ATU), and average treatment effect (ATE) were statistically insignificant for all three outcome variables.

## 4 Discussion

Periodic evaluation of the quality of data is important to ensure the accuracy of inferences drawn from the data (Borkotoky, 2014). Thus, maintaining data quality becomes the fundamental objective in any large sampling survey, so that they produce high-quality data that provide a reasonably close representation of the economic, social, and demographic indicators of the country. With the increasing sample size of NFHS, innovations in survey implementation are necessary to safeguard data quality. It is essential to record the possible outcomes of these innovations on data quality, particularly the introduction of the modular approach in NFHS, which could pave the way for future surveys. This study provides evidence to fill this research gap and several crucial findings on survey-related issues.

This study suggests that the adoption of the modular approach in the survey has an insignificant effect in terms of data collection on the age of women and children and skipping key questions. Although the association between not using contraceptive methods and administration of the state module was marginally significant in the results of the two-level logistic regression model, the results from PSM showed insignificant intervening effects of the administration of the state module on not using contraceptive methods. PSM analysis was performed to check the robustness of our argument and draw conclusions.

One previous study on this subject found that a longer version of the questionnaire was associated with poor data quality (Bradley, 2016). A recently published DHS methodological report that analysed the three DHS surveys using longer and shorter versions of the questionnaire in India (NFHS-4), Kenya (KDHS-2014), and South Africa (2016)found no difference in data quality with different lengths of questionnaires (Allen et al., 2019). These findings indicate that innovation in survey implementation, such as adopting nested design and modular approaches, is an excellent strategy to maintain data quality despite expanding content and coverage in large-scale surveys, providing multiple-level estimates on a wide range of topics. In our study, the effects of introducing the modular approach on data recording were negligible. This is perhaps due to the different innovations mentioned in Supplementary Table S1. For instance, the data collection process has become easier with the help of computer-assisted personal interviews (CAPI) compared to the earlier paper-pencil-based exercise. Field monitoring and checking of real-time data collected in the field helped safeguard the data collection process, even for the longer version of the questionnaire.

Despite the insignificant effects of administration of the longer version of the questionnaire on the recording of the age of women and children and the skipping of the key questions at the individual level, the effect of a higher number of eligible women in a PSU is visible. The results indicate that the administration of the state module itself does not have any effects on the recording of the key outcome indicators; rather, the pressure of a higher number of eligible women along with state module administration at the PSU level leads to skipping the questions. In general, the survey teams allot a certain time (4–5 days for the shorter and longer versions of the questionnaires) to cover the 22 households in a PSU. With an increase in the number of eligible women at the PSU level, particularly in households selected for the state module, the risk of skipping questions on ANC and contraceptive use increased. Therefore, PSUs with a higher number of eligible women should receive more focus from the data quality perspective and may be given more time for data collection to ensure data quality.

The effects of a higher number of eligible women on key skipping questions are significant at both the PSU and household levels. This result indicates that if the number of eligible women increases in a household, the pressure to administer the questionnaire also increases, leading to intentional skipping of key questionnaires to reduce the burden and shorten the duration of the interview.

The effects of the administration of the state module on key outcome indicators were not significant at the individual level, but there were evident state-wise variations in age and birth displacement, skipping questions on ANC, and contraceptive use. These differentials in age, birth displacement, and skipping questions, are the result of regional disparities in demographic and health care indicators. For instance, North Indian states, such as Uttar Pradesh, Bihar, and Madhya Pradesh have a higher level of skipping which indicates a lower level of access to contraceptive use and ANC compared to the South Indian states. This regional variation is also observed in other large-scale sample surveys in India that did not adopt the modular approach in their sample design (IIPS, 2010; India, 2014).

The lower level of access to ANC and contraceptive use did not explain the state-wise differences in age, birth displacement, and age heaping. The recording of these indicators may be influenced by variations in the training of the investigators and the method of data collection in the field across survey agencies. In the current form of data, the effects of survey agencies on skipping key questions, such as ANC and contraceptive use, age and birth displacement, and age heaping, cannot be evaluated. Investigator-level information may be useful for assessing the agency-level effects on the quality of reporting of key indicators. Therefore, conducting a primary survey with interview investigators from different agencies may answer the question of the effects of different agencies on data collection to some extent.

The use of a translator has emerged as an important indicator for skipping questions on ANC and contraceptive use. Perhaps the inclusion of a third person as a translator in the conversation between the investigator and respondents hampers rapport building. (Renschler, 2013). In addition, the misunderstanding of questions by the investigators may generate a non-response or skipping of questions. Thus, the use of a translator may raise concerns about skipping questions, as well as for age and birth reporting.

In addition to the key data quality predictors, this study also indicates that demographic and socioeconomic variables, such as age, level of education, and wealth status of the household, are closely associated with the age heaping of women, birth displacement, and key skipping questions. In line with previous studies, with an increase in the age of women, the reporting of an age ending at 0 and 5 years increases (Lyons-Amos & Stones, 2017). A possible reason for the reporting issue in the older age groups is the higher recall bias among women in the older age group than in the younger age groups. With increasing age, the likelihood of contraceptive use increases as women achieve their desired number of children at a higher age. However, ANC visits decrease with an increase in age, which has also been found in previous studies (Bose & Trent, 2006 (Pandey & Karki, 2014);).

## 5 Conclusions and recommendations

This study offers several conclusions and implications for future research. *First*, the mere administration of the state module as the longer version of the questionnaire is not associated with data quality in the recording of information at the individual level. However, the pressure of eligible women, as well as the administration of the longer version of the questionnaire at the PSU level affects the reporting of the key demographic and health care indicators. Thus, at the PSU level, reducing the pressure on the investigators during data collection would help improve data recording. Therefore, mapping and household listing should also collect auxiliary information, including the number of eligible women during the house-listing process, that would give an idea about the number of eligible women before the survey. This can be achieved by increasing the number of listers in the mapping and household listing teams. Accordingly, the time required to cover eligible women in a PSU may be allotted so that the PSUs with differential workloads get enough time to cover eligible women. *Second,* state-wise variations in birth and age displacement and key skipping questions were evident in this study. This state-wise variation may be partially explained by the difference in training across the agencies of the respective states, for which a primary survey may be conducted to investigate the investigators’ training and data collection strategies across the survey agencies. *Third*, the use of a translator may hamper the quality of recording information from the respondents. Hence, at the agency level, investigators should be selected considering the quality of investigators in terms of their qualifications, communication skills, and language, focusing on the languages spoken in the area/region. The use of translators in urban areas may be lower than in rural areas, as multilingual practices in urban areas may be higher than in rural areas; hence, the focus should be on rural areas. *Fourth*, although the introduction of CAPI has reduced the duration of the interview, the number of questions has been increasing over the subsequent rounds of NFHS. Therefore, it is recommended to add a few more sections to the state module and reduce them from the district module. Some sections of the questionnaire, particularly other health-related issues, may be shifted from the district module to the state module. This would reduce the pressure on the investigator as well as the duration of the interview in the overall framework, adopting a nested design and modular approach.

## Ethics

This study was based on secondary data available in the public domain for research purposes. Therefore, ethical approval was not obtained from the institutional review board.

## Funding

This work was supported by the 10.13039/100000865Bill & Melinda Gates Foundation, Seattle, WA [grant #OPP1194597].

## Financial disclosure statement

This paper was written as part of DataQi project of the 10.13039/100005974Population Council funded by 10.13039/100000865Bill & Melinda Gates Foundation (grant **#** OPP1194597).

## Author statement

Shri Kant Singh: Conceptualization; Validation; Investigation; Writing-Review & Editing; Supervision. Santosh Kumar Sharma: Methodology; Formal Analysis; Writing- Original Draft; Writing-Review & Editing. Md. Juel Rana: Methodology; Formal Analysis; Writing- Original Draft; Writing-Review & Editing; Validation. Akash Porwal: Writing - Review & Editing; Validation. Laxmi Kant Dwivedi: Writing-Review & Editing; Validation; Supervision.

## Declaration of competing interest

The authors declare that they have no conflict of interest.

## Data Availability

Data will be made available on request.
