# Supplementary material for: Effectiveness of modular approach in ensuring data quality in large-scale surveys: Evidence from National Family Health Survey – 4 (2015–2016)
Source: SSM Popul Health. 2022 Oct 4;19:101254. doi: 10.1016/j.ssmph.2022.101254 (PMC9550650; doi:10.1016/j.ssmph.2022.101254)
Supplement: Multimedia component 1 [file mmc1.docx]

**Supplementary File**

**Whipple Index**

The Whipple index was originally computed for population aged 23-62 years. To comply better with the DHS data that provides particular information on women of reproductive ages, we applied the index to ages between 18 and 47 years –as mentioned by Engelhardt (2005) and as shown in the following formula:

Whipple Index (5 year range) = $\frac{\sum{(P}_{20}+P_{25}+P_{30}+P_{35}\ldots\ldots.P_{45})}{\left( \frac{1}{5} \right)\sum{(P}_{18}+P_{19}+P_{20}+P_{21}\ldots\ldots.P_{47})}$ * 100

**Myer’s Index**

The Myer’s index is based on the principle that in the absence of age heaping, the aggregate population of each age, ending in one of the digits 0 to 9, should represent 10% of the total population. The index is calculated by summing the number of people whose age ends with a particular digit for the population aged 10 and over, and then for the population aged 20 and over. Each series is then weighted and the results are added to obtain a blended population. Myers’ blended index is obtained by summing the absolute deviations between the aggregate and theoretical distributions (10%) The range of Myers's index is 0 to 90. The range is further classified into three categories as low (<10), moderate (10–20), and high (>20).

**Multilevel logistic regression model**

A two-level logistic regression model has been applied for three indicators namely, digit preference (age heaping) at 0 and 5, no ANC visits, and not using contraceptive methods. The application of the multilevel modelling was justified by the hierarchal structure of the survey, where women were nested within household, the household were nested within PSUs and PSU’s were nested within states. In these two-level logistic regression analyses, the pressure of state module has been incorporated combining the proportion of the household which have eligible women in a PSU, the average number of eligible women per household in the PSU, and the proportion of women to whom state module is administered. Total three models were fitted in the analysis. In Multilevel analysis, a systemic model building procedure was adopted, and altogether three models were estimated. Model 1 was the null model. In model 1, we have included only to assess the extent of variation in age heaping between communities and the advisability of using a multilevel modeling strategy. Model 2 included individual/household characteristics such as household included in state modules, the number of visits to households, use of the translator, number of eligible women in the household, age group of women, wealth quintile, level of education of women, mass media exposure, working status, religion, and caste. Model 3 included community-level factors such as the pressure of state module questionnaire, place of residence (urban, rural), and states. In this way, estimating the models allowed identifying factors that reduced the significance of each model's variable of interest. Further, likelihood ratio tests were used to compare the goodness-of-fit of the two models. The difference in deviance (-2 log-likelihood) of two nested models has a χ^2^ distribution with degrees of freedom equal to the additional number of predictors in the larger model.

We specified a series of three level random intercept logistic models for the probability of a individual I in HH j, state k had experience of violence (Yijk=1)

Logit (π_ijk_ )= β_o_ + BX_ijk_ + (f_0k_ + v_0jk_ + u_0ij_) …………(1)

This model estimates the log odds of π_ijk_ adjusted for vector (X_ijk_) of above-mentioned independent variables measured at the individual level.

The parameter β_o_ represents the log odds of digit preference (age heaping) at 0 and 5, no ANC visits, and not using contraceptive methods for an individual belonging to the reference category of all the categorical variables. The random effect inside the brackets are interpreted as residual differential for the state k (f_0k_ ), PSU j (v_0jk_ ) and individual I (u_0ij_ ). All three residuals are assumed to be independent and normally distributed with mean 0 and variance σ^2^ f_0_, σ^2^ v_0_ and σ^2^ u_0_ respectively. These variances quantify between states and between PSU variations respectively in the log odds of women with violence on all the individual characteristics. For binary outcome the variance at lowest level cannot be obtained directly from the model and the remaining variance is assumed to simply be a function of the binomial distribution. Based on the variance estimates of random effect, the proportion of variation in the log odds of experience violence to each level is also known as variance partitioning coefficient (VPC) can be calculated.

VPC_z_ = σ^2^_z_/ (σ_f0_^2^ + σ_v0_^2^ +3.29) ……(2)

Here total variation is calculated using latent variable method approach and treated the between individual variation as having a variance of a standard logistic distribution approximated as π2/3=3.29 (Goldstein et al., 2002).

**Supplementary Figure and Tables**

**Figure S1: Kernel density of plots of the propensity score matching between state and district modules in India, 2015-16**


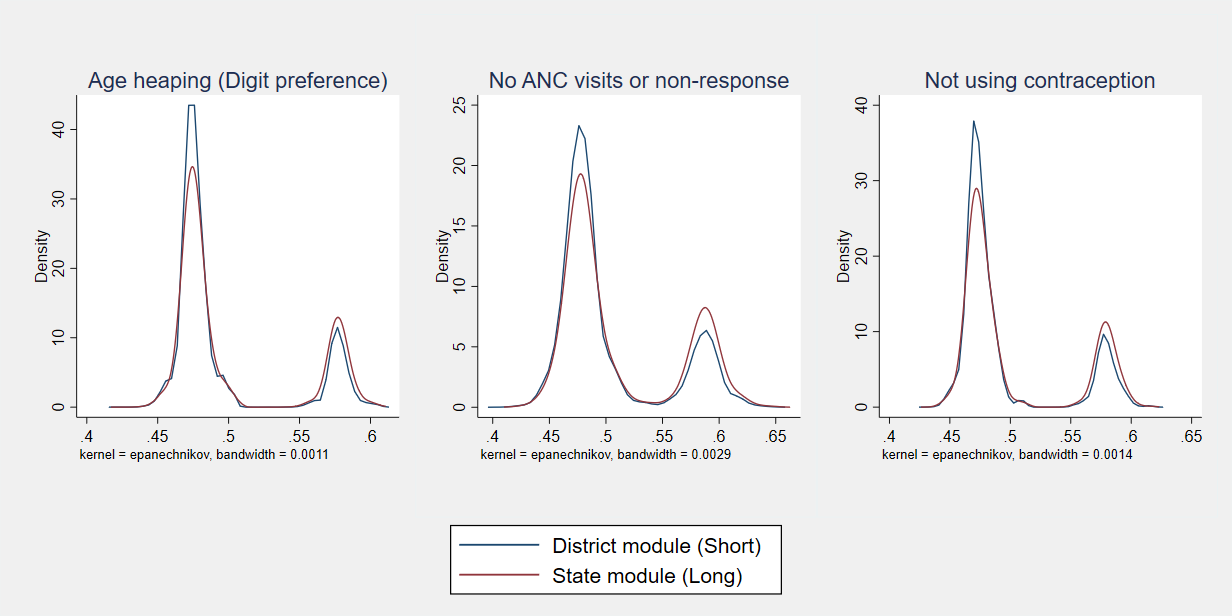


**Table S1: Innovations in survey implementation in NFHS-4, 2015-16**

|  | **Innovations in survey implementation** | **Expected Outcome** |
| --- | --- | --- |
| 1 | Developing nested designs using modular approach | ensure data quality despite expanded contents and coverage of NFHS in India. |
| 2 | Paradigm shift in data collection process with introduction of CAPI | provides an opportunity for back check of information to improve data quality |
| 3 | Minimizing instrumental errors, human errors and transcription errors in collection of biomarkers | ensures the accuracy and consistency of the measurements which will produce high quality of data and will allows comparability with other DHS surveys across the world |
| 4 | Multilayer monitoring and supervision of field work | minimizing the missing events due to communications gap between interviewers and respondents |
| 5 | Developing error messages in the data collected from a PSU by supervisor | ensure internal inconsistency in data with a provision of immediate corrections |
| 6 | Generating POQR on selected indicators before completing any PSU | application of POQR helps in revisiting in a sub sample of interviewed households to ensure accuracy and reliability of information and if there is any problem, go back to interviewer’s CAPI for correcting that information before resynchronizing the data on supervisor’s CAPI. |
| 7 | Real time access to data using field check tables an opportunity for immediate feedback and also through skype interactions | tracking those underperforming teams/interviewers and motivating them with examples of others |

| **Table S2: Descriptive statistics of the PSUs in terms of response to longer version of questionnaire across the states of India, 2015-16** | | | | | | | |
| --- | --- | --- | --- | --- | --- | --- | --- |
| States | Number of PSU | Mean | Standard Deviation | Minimum | Maximum | <45% | >55% |
| Andaman and Nicobar Islands | 37 | 50.3 | 6.9 | 35.0 | 64.0 | 21.6 | 29.7 |
| Andhra Pradesh | 161 | 50.8 | 8.5 | 25.0 | 76.2 | 24.2 | 29.8 |
| Arunachal Pradesh | 231 | 51.5 | 8.8 | 23.8 | 78.6 | 21.7 | 36.4 |
| Assam | 374 | 49.8 | 7.1 | 31.3 | 71.4 | 23.3 | 19.8 |
| Bihar | 537 | 49.4 | 7.5 | 22.2 | 75.0 | 27.2 | 22.0 |
| Chandigarh | 13 | 47.4 | 8.5 | 35.0 | 64.7 | 38.5 | 15.4 |
| Chhattisgarh | 300 | 50.6 | 8.1 | 27.8 | 81.8 | 21.7 | 28.0 |
| Dadra and Nagar Haveli | 18 | 46.6 | 7.7 | 35.7 | 65.2 | 38.9 | 16.7 |
| Daman and Diu | 40 | 52.3 | 11.7 | 25.0 | 80.0 | 25.0 | 45.0 |
| Goa | 76 | 48.9 | 8.7 | 30.0 | 71.4 | 30.3 | 18.4 |
| Gujarat | 518 | 49.7 | 9.1 | 10.0 | 81.8 | 30.5 | 26.6 |
| Haryana | 256 | 48.1 | 6.8 | 25.0 | 65.0 | 32.4 | 14.8 |
| Himachal Pradesh | 260 | 50.3 | 10.1 | 20.0 | 85.7 | 28.9 | 29.2 |
| Jammu and Kashmir | 485 | 50.9 | 7.2 | 26.7 | 71.0 | 20.0 | 27.2 |
| Jharkhand | 391 | 49.8 | 7.8 | 26.1 | 71.0 | 27.1 | 28.1 |
| Karnataka | 364 | 50.3 | 7.9 | 26.3 | 74.1 | 24.5 | 26.7 |
| Kerala | 232 | 49.3 | 9.1 | 21.7 | 78.6 | 29.7 | 21.6 |
| Lakshadweep | 14 | 50.9 | 9.0 | 29.4 | 65.6 | 7.1 | 35.7 |
| Madhya Pradesh | 765 | 49.8 | 8.3 | 20.6 | 73.9 | 26.9 | 27.1 |
| Maharashtra | 426 | 49.1 | 9.3 | 21.4 | 77.3 | 29.8 | 22.5 |
| Manipur | 173 | 49.8 | 8.1 | 23.5 | 83.3 | 25.4 | 24.9 |
| Meghalaya | 110 | 49.5 | 7.6 | 26.3 | 73.7 | 23.6 | 21.8 |
| Mizoram | 165 | 51.4 | 9.1 | 28.6 | 76.0 | 23.6 | 32.1 |
| Nagaland | 166 | 50.8 | 9.7 | 29.4 | 85.7 | 24.1 | 28.9 |
| Delhi | 95 | 51.5 | 13.2 | 18.8 | 82.4 | 32.6 | 40.0 |
| Odisha | 452 | 49.9 | 7.9 | 28.6 | 73.1 | 26.6 | 25.7 |
| Puducherry | 64 | 49.8 | 9.3 | 19.2 | 74.1 | 35.9 | 29.7 |
| Punjab | 243 | 48.9 | 8.1 | 27.8 | 78.6 | 30.0 | 22.6 |
| Rajasthan | 93 | 49.0 | 8.0 | 28.6 | 69.2 | 25.8 | 22.6 |
| Sikkim | 75 | 50.1 | 7.4 | 32.1 | 70.0 | 22.7 | 26.7 |
| Tamil Nadu | 513 | 50.2 | 8.5 | 18.8 | 80.0 | 25.5 | 28.3 |
| Tripura | 88 | 48.5 | 8.6 | 15.4 | 64.0 | 34.1 | 22.7 |
| Uttar Pradesh | 1,145 | 49.6 | 8.8 | 16.7 | 100 | 30.6 | 26.0 |
| Uttarakhand | 230 | 50.1 | 8.0 | 30.4 | 78.9 | 23.0 | 23.0 |
| West Bengal | 232 | 49.8 | 7.7 | 22.2 | 75.0 | 27.6 | 23.3 |
| Telangana | 124 | 50.3 | 8.2 | 29.2 | 70.6 | 25.8 | 28.2 |
| India | 9,466 | 49.9 | 8.4 | 10.0 | 100 | 26.9 | 25.9 |
| Note: Percentage of PSUs which have less than 45% and more than 55% of households with state module administered are presented in the last two column | | | | | | | |

| **Table S3: Whipple index by shorter and longer version of the questionnaire across the states of India, 2015-16** | | | | |
| --- | --- | --- | --- | --- |
| States | Whipple Index | | Sample | |
|  | Short | Long | Short | Long |
| Andaman and Nicobar Islands | 1.18 | 1.10 | 408 | 394 |
| Andhra Pradesh | 1.51 | 1.51 | 1490 | 1544 |
| Arunachal Pradesh | 1.69 | 1.66 | 2009 | 2084 |
| Assam | 1.42 | 1.44 | 3997 | 4029 |
| Bihar | 1.70 | 1.67 | 6313 | 6230 |
| Chandigarh | 1.13 | 1.35 | 119 | 111 |
| Chhattisgarh | 1.33 | 1.28 | 3413 | 3446 |
| Dadra and Nagar Haveli | 0.77 | 1.21 | 169 | 141 |
| Daman and Diu | 1.39 | 1.34 | 291 | 309 |
| Goa | 1.04 | 1.10 | 766 | 716 |
| Gujarat | 1.23 | 1.27 | 5410 | 5292 |
| Haryana | 1.34 | 1.36 | 3145 | 2898 |
| Himachal Pradesh | 1.04 | 0.97 | 2495 | 2520 |
| Jammu and Kashmir | 1.38 | 1.34 | 6035 | 6152 |
| Jharkhand | 1.57 | 1.56 | 4003 | 3963 |
| Karnataka | 1.35 | 1.30 | 3794 | 3830 |
| Kerala | 1.23 | 1.11 | 2119 | 2085 |
| Lakshadweep | 0.98 | 1.02 | 178 | 187 |
| Madhya Pradesh | 1.44 | 1.39 | 8555 | 8387 |
| Maharashtra | 1.19 | 1.22 | 4239 | 4105 |
| Manipur | 1.29 | 1.27 | 1846 | 1795 |
| Meghalaya | 1.47 | 1.43 | 1257 | 1209 |
| Mizoram | 1.19 | 1.14 | 1587 | 1689 |
| Nagaland | 1.32 | 1.29 | 1397 | 1466 |
| Delhi | 1.09 | 1.41 | 766 | 774 |
| Odisha | 1.35 | 1.27 | 4704 | 4712 |
| Puducherry | 1.09 | 1.33 | 732 | 709 |
| Punjab | 1.26 | 1.22 | 2731 | 2656 |
| Rajasthan | 1.41 | 1.44 | 5667 | 5595 |
| Sikkim | 1.29 | 1.27 | 816 | 800 |
| Tamil Nadu | 1.30 | 1.26 | 5257 | 5285 |
| Tripura | 1.20 | 1.27 | 869 | 820 |
| Uttar Pradesh | 1.43 | 1.41 | 12871 | 12815 |
| Uttarakhand | 1.31 | 1.37 | 2302 | 2270 |
| West Bengal | 1.32 | 1.31 | 2436 | 2429 |
| Telangana | 1.40 | 1.50 | 1133 | 1138 |
| India | 1.37 | 1.36 | 105319 | 104585 |
| Note: The Whipple index value more than 1.05 indicates the digit preference for 0 and 5; The women aged 18 to 47 years are included in these analyses | | | | |

| **Table S4: Myers blended index by shorter and longer version of the questionnaire across the states of India, 2015-16** | | | | | |
| --- | --- | --- | --- | --- | --- |
| States | Myers Index | | Sample | | |
|  | Short | Long | | Short | Long |
| Andaman and Nicobar Islands | 5.6 | 8.0 | | 400 | 383 |
| Andhra Pradesh | 14.7 | 12.0 | | 1484 | 1502 |
| Arunachal Pradesh | 15.8 | 15.3 | | 1944 | 2047 |
| Assam | 10.6 | 11.8 | | 3849 | 3872 |
| Bihar | 18.6 | 18.0 | | 5924 | 5840 |
| Chandigarh | 6.8 | 16.3 | | 117 | 111 |
| Chhattisgarh | 10.4 | 9.6 | | 3291 | 3327 |
| Dadra and Nagar Haveli | 14.3 | 14.1 | | 157 | 139 |
| Daman and Diu | 12.3 | 11.9 | | 276 | 303 |
| Goa | 3.8 | 4.6 | | 745 | 716 |
| Gujarat | 5.8 | 6.7 | | 5194 | 5066 |
| Haryana | 11.9 | 10.9 | | 3023 | 2790 |
| Himachal Pradesh | 5.0 | 3.5 | | 2446 | 2495 |
| Jammu and Kashmir | 10.3 | 10.1 | | 5707 | 5872 |
| Jharkhand | 15.6 | 14.9 | | 3799 | 3732 |
| Karnataka | 9.1 | 7.3 | | 3685 | 3706 |
| Kerala | 8.4 | 6.3 | | 2132 | 2047 |
| Lakshadweep | 8.5 | 4.8 | | 175 | 189 |
| Madhya Pradesh | 12.9 | 10.9 | | 8173 | 8094 |
| Maharashtra | 6.2 | 6.8 | | 4053 | 3955 |
| Manipur | 7.8 | 8.0 | | 1814 | 1753 |
| Meghalaya | 13.3 | 12.2 | | 1215 | 1172 |
| Mizoram | 5.5 | 5.7 | | 1549 | 1616 |
| Nagaland | 9.6 | 8.0 | | 1364 | 1427 |
| Delhi | 9.2 | 9.7 | | 743 | 739 |
| Odisha | 7.0 | 5.4 | | 4589 | 4579 |
| Puducherry | 6.3 | 8.8 | | 729 | 720 |
| Punjab | 8.1 | 7.4 | | 2701 | 2588 |
| Rajasthan | 13.3 | 12.8 | | 5359 | 5304 |
| Sikkim | 10.9 | 11.7 | | 786 | 789 |
| Tamil Nadu | 9.0 | 7.9 | | 5213 | 5242 |
| Tripura | 9.0 | 9.2 | | 844 | 795 |
| Uttar Pradesh | 13.0 | 13.3 | | 12052 | 11999 |
| Uttarakhand | 9.2 | 10.2 | | 2186 | 2187 |
| West Bengal | 9.5 | 7.8 | | 2365 | 2355 |
| Telangana | 11.0 | 14.2 | | 1086 | 1111 |
| India | 9.8 | 9.3 | | 101169 | 100562 |
| Note: The women aged 20 years to 49 years have been included in this exercise | | | | | |

| **Table S5: Age displacement from 15 years to 14 years and from 49 years to 50 years by shorter and longer version of questionnaire across the states of India, 2015-16** | | | | | | | | |
| --- | --- | --- | --- | --- | --- | --- | --- | --- |
| States | 14/15 years | | | | 50/49 years | | | |
|  | Ratio | | Sample | | Ratio | | Sample | |
|  | Short | Long | Short | Long | Short | Long | Short | Long |
| Andaman and Nicobar Islands | 0.92 | 0.56 | 25 | 28 | 0.50 | 1.00 | 6 | 10 |
| Andhra Pradesh | 1.74 | 1.31 | 96 | 113 | 0.55 | 0.81 | 68 | 58 |
| Arunachal Pradesh | 1.12 | 0.93 | 191 | 201 | 1.43 | 1.00 | 97 | 118 |
| Assam | 1.00 | 0.98 | 344 | 309 | 1.06 | 1.10 | 134 | 124 |
| Bihar | 0.80 | 0.66 | 730 | 671 | 1.31 | 1.43 | 222 | 187 |
| Chandigarh | 0.43 | 8.00 | 10 | 9 | 0.25 | 1.00 | 5 | 2 |
| Chhattisgarh | 0.49 | 0.66 | 266 | 297 | 0.51 | 0.28 | 103 | 95 |
| Dadra and Nagar Haveli | 0.67 | 1.25 | 10 | 9 | ─ | 1.00 | 3 | 8 |
| Daman and Diu | 2.14 | 1.00 | 22 | 26 | 4.00 | 2.25 | 15 | 13 |
| Goa | 1.06 | 1.00 | 35 | 58 | 1.64 | 0.47 | 37 | 22 |
| Gujarat | 0.85 | 1.01 | 419 | 399 | 1.65 | 1.41 | 215 | 212 |
| Haryana | 0.98 | 1.23 | 192 | 210 | 0.78 | 0.91 | 105 | 84 |
| Himachal Pradesh | 1.00 | 0.95 | 190 | 179 | 0.85 | 0.62 | 85 | 94 |
| Jammu and Kashmir | 1.09 | 0.83 | 456 | 467 | 0.74 | 0.43 | 157 | 195 |
| Jharkhand | 0.95 | 0.78 | 358 | 387 | 1.43 | 1.18 | 141 | 124 |
| Karnataka | 1.09 | 0.92 | 295 | 277 | 1.58 | 1.36 | 186 | 177 |
| Kerala | 1.18 | 1.19 | 157 | 158 | 1.05 | 1.04 | 127 | 96 |
| Lakshadweep | 1.40 | 1.57 | 12 | 18 | 2.00 | 1.00 | 9 | 6 |
| Madhya Pradesh | 0.83 | 0.73 | 768 | 744 | 0.69 | 0.66 | 293 | 300 |
| Maharashtra | 0.94 | 0.95 | 334 | 321 | 1.87 | 1.68 | 198 | 185 |
| Manipur | 0.94 | 0.94 | 153 | 155 | 0.49 | 0.51 | 64 | 56 |
| Meghalaya | 1.08 | 0.93 | 181 | 158 | 1.65 | 1.38 | 82 | 76 |
| Mizoram | 1.03 | 1.08 | 148 | 158 | 1.25 | 1.59 | 63 | 75 |
| Nagaland | 1.02 | 1.46 | 109 | 138 | 1.54 | 1.43 | 66 | 56 |
| Delhi | 0.75 | 0.76 | 49 | 67 | 0.93 | 0.93 | 29 | 27 |
| Odisha | 0.78 | 0.86 | 365 | 389 | 0.57 | 0.56 | 180 | 176 |
| Puducherry | 1.09 | 0.48 | 46 | 37 | 0.74 | 0.41 | 33 | 38 |
| Punjab | 0.87 | 1.25 | 168 | 155 | 0.93 | 1.00 | 89 | 82 |
| Rajasthan | 0.91 | 0.75 | 494 | 500 | 0.99 | 0.88 | 137 | 126 |
| Sikkim | 0.59 | 0.68 | 46 | 52 | 1.38 | 0.81 | 19 | 29 |
| Tamil Nadu | 0.72 | 0.74 | 343 | 334 | 0.84 | 0.68 | 213 | 224 |
| Tripura | 0.97 | 0.74 | 57 | 59 | 0.79 | 0.71 | 34 | 24 |
| Uttar Pradesh | 0.74 | 0.85 | 1390 | 1349 | 0.89 | 0.89 | 357 | 320 |
| Uttarakhand | 0.93 | 0.91 | 220 | 241 | 1.06 | 0.70 | 66 | 63 |
| West Bengal | 0.94 | 0.74 | 186 | 191 | 0.49 | 0.51 | 79 | 68 |
| Telangana | 1.28 | 1.15 | 91 | 88 | 1.05 | 1.32 | 45 | 44 |
| India | 0.88 | 0.86 | 8956 | 8952 | 1.00 | 0.89 | 3762 | 3594 |
| Note: Ratio between reported number of women at 14 years and 15 years and by number of women at 50 years and 49 years are presented | | | | | | | | |

| **Table S6: Birth displacement from 5 years to 6 years by shorter and longer version of questionnaire across the states of India, 2015-16** | | | | |
| --- | --- | --- | --- | --- |
| States | Ratio | | Sample | |
|  | Short | Long | Short | Long |
| Andaman and Nicobar Islands | 1.64 | 0.73 | 37 | 52 |
| Andhra Pradesh | 0.85 | 0.81 | 202 | 183 |
| Arunachal Pradesh | 1.55 | 1.38 | 382 | 416 |
| Assam | 1.04 | 1.07 | 626 | 655 |
| Bihar | 1.01 | 0.95 | 1687 | 1747 |
| Chandigarh | 1.00 | 2.80 | 16 | 19 |
| Chhattisgarh | 0.76 | 0.81 | 582 | 595 |
| Dadra and Nagar Haveli | 0.89 | 1.14 | 17 | 30 |
| Daman and Diu | 1.10 | 0.68 | 42 | 37 |
| Goa | 1.57 | 0.96 | 77 | 98 |
| Gujarat | 1.02 | 1.04 | 822 | 814 |
| Haryana | 0.96 | 0.89 | 497 | 449 |
| Himachal Pradesh | 1.26 | 1.07 | 352 | 354 |
| Jammu and Kashmir | 1.09 | 1.13 | 945 | 929 |
| Jharkhand | 1.13 | 1.25 | 823 | 871 |
| Karnataka | 1.15 | 1.31 | 525 | 535 |
| Kerala | 1.10 | 0.82 | 239 | 207 |
| Lakshadweep | 2.50 | 1.00 | 14 | 20 |
| Madhya Pradesh | 0.95 | 0.82 | 1517 | 1611 |
| Maharashtra | 1.07 | 1.19 | 641 | 619 |
| Manipur | 1.28 | 0.93 | 328 | 342 |
| Meghalaya | 1.12 | 1.27 | 301 | 327 |
| Mizoram | 1.13 | 1.05 | 323 | 312 |
| Nagaland | 1.10 | 1.01 | 294 | 289 |
| Delhi | 0.95 | 0.89 | 111 | 85 |
| Odisha | 1.05 | 0.99 | 689 | 688 |
| Puducherry | 0.87 | 1.10 | 88 | 103 |
| Punjab | 1.03 | 1.08 | 310 | 324 |
| Rajasthan | 1.08 | 1.06 | 1038 | 1081 |
| Sikkim | 0.97 | 1.11 | 69 | 76 |
| Tamil Nadu | 0.83 | 0.86 | 603 | 675 |
| Tripura | 1.12 | 1.06 | 108 | 111 |
| Uttar Pradesh | 1.05 | 1.09 | 2765 | 2710 |
| Uttarakhand | 1.01 | 0.89 | 384 | 373 |
| West Bengal | 1.02 | 0.96 | 337 | 341 |
| Telangana | 0.91 | 1.01 | 168 | 167 |
| India | 1.04 | 1.02 | 17959 | 18245 |
| Note: Ratio between reported number of children aged 6 years and 5 years are presented | | | | |

| **Table S7: Percentage of no ANC visits and no contraceptive use by shorter and longer version of questionnaire across the states of India, 2015-16** | | | | | | | | | |
| --- | --- | --- | --- | --- | --- | --- | --- | --- | --- |
| States | No ANC | | | | No contraceptive use | | | | |
|  | Percentage | | Sample | | Percentage | | Sample | |  |
|  | Short | Long | Short | Long | Short | Long | Short | Long |  |
| Andaman and Nicobar Islands | 7.8 | 3.5 | 90 | 86 | 43.8 | 42.8 | 331 | 318 |  |
| Andhra Pradesh | 1.6 | 0.3 | 380 | 376 | 32.7 | 30.7 | 1313 | 1313 |  |
| Arunachal Pradesh | 46.8 | 46.1 | 624 | 651 | 71.5 | 70.5 | 1656 | 1713 |  |
| Assam | 8.9 | 9.4 | 1343 | 1374 | 45.3 | 43.6 | 3359 | 3291 |  |
| Bihar | 40.9 | 41.2 | 2728 | 2779 | 72.0 | 70.9 | 5841 | 5734 |  |
| Chandigarh | 3.3 | 12.0 | 30 | 25 | 22.2 | 23.6 | 90 | 89 |  |
| Chhattisgarh | 2.9 | 3.4 | 1100 | 1109 | 42.1 | 41.9 | 2746 | 2774 |  |
| Dadra and Nagar Haveli | 10.3 | 10.0 | 58 | 60 | 55.9 | 64.8 | 143 | 128 |  |
| Daman and Diu | 6.8 | 18.9 | 73 | 90 | 56.7 | 66.3 | 224 | 240 |  |
| Goa | 5.6 | 3.2 | 160 | 187 | 75.8 | 75.1 | 537 | 558 |  |
| Gujarat | 17.7 | 15.6 | 1558 | 1561 | 52.7 | 54.1 | 4590 | 4525 |  |
| Haryana | 15.3 | 18.7 | 921 | 852 | 31.4 | 30.6 | 2659 | 2438 |  |
| Himachal Pradesh | 9.3 | 8.7 | 626 | 679 | 39.4 | 39.6 | 2083 | 2159 |  |
| Jammu and Kashmir | 8.4 | 8.8 | 1807 | 1856 | 43.0 | 42.9 | 4350 | 4491 |  |
| Jharkhand | 22.9 | 22.4 | 1475 | 1488 | 58.9 | 59.3 | 3468 | 3445 |  |
| Karnataka | 8.1 | 8.4 | 948 | 978 | 45.3 | 46.5 | 3119 | 3130 |  |
| Kerala | 7.5 | 8.2 | 454 | 503 | 47.2 | 47.3 | 1796 | 1759 |  |
| Lakshadweep | 6.4 | 10.2 | 47 | 49 | 72.2 | 71.9 | 144 | 153 |  |
| Madhya Pradesh | 21.2 | 23.1 | 2739 | 2713 | 47.7 | 46.6 | 7511 | 7391 |  |
| Maharashtra | 7.1 | 8.1 | 1191 | 1134 | 32.9 | 31.9 | 3565 | 3449 |  |
| Manipur | 12.4 | 10.0 | 669 | 667 | 75.7 | 74.7 | 1369 | 1348 |  |
| Meghalaya | 17.7 | 14.5 | 514 | 509 | 74.5 | 73.4 | 912 | 880 |  |
| Mizoram | 11.8 | 11.3 | 557 | 558 | 64.8 | 63.4 | 1013 | 1111 |  |
| Nagaland | 55.2 | 55.0 | 491 | 484 | 74.4 | 71.3 | 1017 | 1034 |  |
| Delhi | 10.9 | 7.9 | 193 | 189 | 51.3 | 51.5 | 596 | 581 |  |
| Odisha | 5.8 | 5.2 | 1420 | 1502 | 38.7 | 38.4 | 3907 | 3831 |  |
| Puducherry | 2.6 | 4.6 | 195 | 195 | 41.8 | 45.0 | 600 | 613 |  |
| Punjab | 3.0 | 2.1 | 631 | 680 | 23.5 | 24.4 | 2205 | 2129 |  |
| Rajasthan | 13.2 | 13.8 | 1789 | 1864 | 38.9 | 39.0 | 4906 | 4884 |  |
| Sikkim | 10.1 | 7.0 | 148 | 171 | 46.8 | 49.6 | 581 | 605 |  |
| Tamil Nadu | 9.5 | 9.9 | 1227 | 1398 | 47.4 | 48.5 | 4351 | 4479 |  |
| Tripura | 11.0 | 8.8 | 209 | 262 | 34.8 | 31.0 | 755 | 723 |  |
| Uttar Pradesh | 21.3 | 21.9 | 4532 | 4660 | 51.9 | 52.0 | 10598 | 10483 |  |
| Uttarakhand | 20.6 | 18.9 | 708 | 688 | 40.4 | 40.5 | 1880 | 1851 |  |
| West Bengal | 9.8 | 9.5 | 706 | 739 | 29.0 | 27.7 | 2243 | 2208 |  |
| Telangana | 2.3 | 3.3 | 265 | 306 | 41.3 | 39.3 | 938 | 953 |  |
| India | 17.0 | 17.1 | 32606 | 33422 | 48.0 | 47.8 | 87396 | 86811 |  |
| Note: No ANC also includes the non-response of the question | | | | | | | | | |

| **Table S8: Results from the propensity score matching: Average treatment on the treated (ATT), average treatment on the untreated (ATU) and average treatment effect (ATE) of digit preference, no ANC visit and not using contraception in India, 2015-16** | | | | | |
| --- | --- | --- | --- | --- | --- |
| Outcome sample | Treated | Controls | Difference | Standard error | T stats |
| **(A) Digit preference – 0 and 5** |  |  |  |  |  |
| Unmatched | 0.2717 | 0.2744 | -0.0027 | 0.0019 | 1.41 |
| ATT | 0.2717 | 0.2747 | -0.0031 | 0.0026 | 1.18 |
| ATU | 0.2744 | 0.2724 | -0.0020 | ─ | ─ |
| ATE | ─ | ─ | -0.0025 | ─ | ─ |
| **(B) No ANC visit and non-response** | |  |  |  |  |
| Unmatched | 0.1705 | 0.1703 | 0.0002 | 0.0029 | 0.08 |
| ATT | 0.1705 | 0.1737 | -0.0032 | 0.0039 | 0.82 |
| ATU | 0.1703 | 0.1698 | -0.0005 | ─ | ─ |
| ATE | ─ | ─ | -0.0019 | ─ | ─ |
| **(C) Not using contraception** |  |  |  |  |  |
| Unmatched | 0.4781 | 0.4795 | -0.0014 | 0.0024 | 0.58 |
| ATT | 0.4781 | 0.4811 | -0.0030 | 0.0032 | 0.94 |
| ATU | 0.4795 | 0.4789 | -0.0006 | ─ | ─ |
| ATE | ─ | ─ | -0.0018 | ─ | ─ |
| Note: Multiple visits of the interviewer to the household, use of translator, presence of more than one eligible women in the household, age in years, wealth status (percentile), years of schooling, access to mass media, religion, caste, residence and states have been included in the list of independent variables in estimating propensity score.  The propensity score matching between district and state module for three separate outcome variables are presented in the Appendix Figure 1. | | | | | |
